# Microwave Heating as an Alternative Quarantine Method for Disinfestation of Stored Food Grains

**DOI:** 10.1155/2013/926468

**Published:** 2013-04-22

**Authors:** Ipsita Das, Girish Kumar, Narendra G. Shah

**Affiliations:** ^1^Department of Electrical Engineering, Indian Institute of Technology, Bombay, Powai, Mumbai 400076, India; ^2^Centre for Technology Alternatives for Rural Areas, Indian Institute of Technology, Bombay, Powai, Mumbai 400076, India

## Abstract

Insects and pests constitute a major threat to food supplies all over the world. Some estimates put the loss of food grains because of infestation to about 40% of the world production. Contemporary disinfestation methods are chemical fumigation, ionizing radiation, controlled atmosphere, conventional hot air treatment, and dielectric heating, that is, radio frequency and microwave energy, and so forth. Though chemical fumigation is being used extensively in stored food grains, regulatory issues, insect resistance, and environmental concerns demand technically effective and environmentally sound quarantine methods. Recent studies have indicated that microwave treatment is a potential means of replacing other techniques because of selective heating, pollution free environment, equivalent or better quality retention, energy minimization, and so forth. The current paper reviews the recent advances in Microwave (MW) disinfestation of stored food products and its principle and experimental results from previous studies in order to establish the usefulness of this technology.

## 1. Importance of Disinfestation

Agricultural commodities produced on the fields have to undergo a series of operations such as harvesting, threshing, winnowing, bagging, transportation, storage, and processing before they reach the consumer, and there are appreciable losses in crop output at all these stages. Various estimates have been made to assess the postharvest food grain losses. The losses are caused either by environmental factors such as temperature, moisture, and type of storage structure or by biological agents, namely, insects, rodents, birds, and fungi. The major losses during production, storage and marketing of food grain are being attributed to infestation by insect pests, microbiological contamination, and physiological changes. Insect infestations can occur just prior to harvest or during storage or in-transit in a variety of carriers. The occurrence and numbers of stored grain insect pests are directly related to geographical and climatic conditions [1]. Almost all species have remarkably high rates of multiplication and, within one season, may destroy 10–15% of the grain and contaminate the rest with undesirable odors and flavors. Insect pests also play a pivotal role in transportation of storage fungi [2]. Therefore, preventing economic losses caused by stored-product insects is important from the field to the consumer's table [3]. The losses during storage are classified as quantity losses and quality losses. Quantity losses occur when the grain is consumed by insects, rodents, mites, birds and microorganisms. Quality losses are reflected as reduced economic value of the crop. The stored-grain insects affect not only the quantity of grain but also the quality of grain. Infestation causes decreased nutritional value, reduced seed germination, and lower economic value and also causes changes in chemical compositions such as increase in moisture, free fatty acid levels, nonprotein nitrogen content, and decrease in pH and protein contents in food grain [4]. They reduce the product quality directly by damage through feeding and indirectly by producing webbing and frass [5]. Also the grain quality has been found to decrease with time with increasing levels of infestation [6]. It is estimated that more than 20,000 species of field and storage pests destroy approximately one-third of the world's food production, valued annually at more than 100 billion dollar [7]. The quantitative and qualitative damage to stored grains and grain product from the insect pests may amount to 20–30% in the tropical zone and 5–10% in the temperate zone [8]. Food grain production in India has reached 250 million tons in the year 2010-2011, in which nearly 20–25% food grains are damaged by stored grain insect pests [7]. With population growth and the amount of cultivatable land shrinking, grain losses will continue to be a problem in the developing countries. Control of stored-product pests is one of the major tasks because the damage inflicted to foodstuff is irreversible. Also with progressive increase in the quantity of food grains and necessity for longer storage periods, these losses will escalate unless disinfestation measures are improved. International organizations such as FDA [9] and FGIS [10] have set tolerances and grade standards regulating the number of insects and insect fragments above specified tolerances to make the product illegal for human consumption. In developed countries even the mere presence of a few insects in a bulk, at densities of considerably less than one insect per kg grain, can cause a serious loss in its market value. In some developed countries grain can be downgraded or rejected completely if even a single live insect is found [11]. The efficient control and removal of stored grain pests from food commodities have long been the goal of entomologists throughout the world. Various methods of insect control have been practiced to save the grain. In recent years, technology has made marked progress in the study of disinfestation of stored-grain insects and of finding improved ways to control them especially to detect latent forms of infestation. The implementation of an insect-disinfestation method requires detailed analysis of all the elements of an infestation problem: the insects, their age, species, and distribution and their survival and developmental rates under different environmental conditions [12]. Conventional chemicals, grain protectants, and fumigants are extensively used around the world to control insect pests in stored commodities because of low cost, fast processing, and easy application. Greater regulation and restriction of methyl bromide use will likely increase the cost of the fumigant, as well as reduce its availability [13]. With the concerns about health hazards of chemical pesticides and their resulting environmental pollution, there is interest in developing alternative, nonchemical process protocol to control insect pests while retaining acceptable product quality. These include conventional hot air or water heating, controlled atmosphere, and dielectric heating (radio frequency (RF) and microwave (MW)). Currently, the use of chemical fumigation remains widespread and the efficient use of RF and MW methods for disinfestation is still in research stage. The current paper reviews the various disinfestation methods of stored food grains with special emphasis on recent advances in microwave disinfestation of stored food grains. The principle of microwave disinfestation, experimental results of quality characteristics of microwave-treated grains, and the challenges of microwave disinfestation have also been described.

## 2. Various Methods of Insect Control

The control of stored-product insects is very important if the quality of foodstuff is to be maintained. The goal of the control measure is to render the habitat unsuitable for the growth and reproduction of stored-product insects. The five major potential quarantine treatment methods used for disinfestation of several insect pests for both the domestic and international markets are chemical fumigation, ionizing radiation, controlled atmosphere, conventional hot air/water heating, and dielectric heating using radio frequency (RF) and microwave (MW) energy which have been described in this paper.

### 2.1. Chemical Method

Since the 1950s, chemical insecticides have been used extensively in grain storage facilities to control stored-product insect pests due to low cost, fast speed in processing, and ease of use. Most postharvest pest management programs, therefore, rely heavily on fumigants. Currently, over 2.5 million tons of chemicals, worth over 30 billion US dollars, are applied to crops in the world [14]. The chemicals used to control insects in the bulk stored-grains are composed of two classes, namely, contact insecticides and fumigants. Contact insecticides, such as malathion, chlorpyrifos-methyl, or deltamethrin, are sprayed directly on grain or structures which provide protection from infestation for several months [15]. Fumigants are gaseous insecticides applied to control insects in grains that are inaccessible by contact insecticide. Some of the commonly used fumigants are methyl bromide (MeBr) and phosphine which rapidly kill all life stages of stored-product insects in food product [16]. Methyl bromide is now under the threat of withdrawal because it apparently depletes the Earth's ozone layer [17]. It has already been established that the use of MeBr has led to serious environmental damage and hazards to people's health. For these reasons, the Montreal Protocol constituted by the United Nations Environment Programme agreed on a phasing out of methyl bromide in the developed countries by 2005 and in the developing countries by 2015 [18]. Phosphine has been used as a replacement of methyl bromide for a long time [19]. Conventional use of phosphine has failed frequently to control insects [20]. Another limiting factor is that insects develop resistance to some particular chemical fumigants. Some of the contact insecticides have become ineffective because of wide-spread resistance in insect populations. A worldwide survey of stored-product insects revealed that 87% of 505 strains of the red flour beetle, *Tribolium castaneum*, collected from 78 countries were resistant to malathion [15]. Resistance to malathion is widespread in Canada, USA, and Australia [21] while resistance to phosphine is great in Australia and India, which may cause control failures [22]. The other major problem associated with the chemical methods is that, even if they are applied with care and in limited quantity, there is a possibility that these chemicals may remain in the food grains and have adverse effects on humans. Fumigation often only kills live larvae or adult insects but does not sterilize the eggs which are still alive in the grain kernels [23]. The consumption of organic products is also increasing each year. There is, therefore, interest in developing an alternative, nonchemical process method to control insect pests in food grains so as to minimize the environmental hazards associated with chemical insecticides while retaining acceptable product quality. 

### 2.2. Ionizing Radiation

It is a process where infested food products are being exposed to ionizing radiation so as to sterilize, kill, or prevent emergence of insect pests by damaging their DNA. Three types of ionizing radiation used on foods are gamma rays from radioactive cobalt-60 and cesium-137, high energy electrons, and X-rays [24]. Irradiation with high energy electrons is usually safer and easier to work with because it can be turned on and off while an isotope is always radiating and humans must be shielded from it [25]. The ability of gamma rays to deeply penetrate pallet loads of food makes it one of the most commonly used in postharvest pest control. Sterilization of many species of insects can be accomplished at lower doses. Rusty grain beetles are sterilized at only 0.6 kGy but saw-toothed grain beetles and red flour beetles require a 2.0 kGy dose [26]. The grain mite, however, requires a much higher dose of 4.5 kGy. Though irradiation doses of 3–5 kGy were reported to be effective in controlling insects, they cause damage to product quality [26]. According to Hasan and Khan [27], high dosage of ionizing radiation has a risk of vitamin loss from the food product. The major drawback is substantial initial investment to establish the facility. In order to be economically feasible, the facility must remain in continuous operation. However, the seasonal nature of food produce prevents efficient use of facilities. Besides, this method has not received wide recognition because of high power consumption, large weight, and overall sizes of the installation. Consumers also have concerns over the disposal of radioactive wastes, the safety of the irradiation technology, and its effect on food [28]. Irradiation treatments often lead to live insects found by inspectors or consumers in the treated product because the applied doses do not immediately kill treated insects [29, 30]. Grains will continue to appear to be infested and a grain buyer cannot be certain that the insects are sterilized. Following irradiation with gamma rays at 0.5 kGy, complete insect mortality occurs in 14 days for rusty grain beetles, 28 days for red flour beetles, 70 days for saw-toothed grain beetles, and 200 days for grain mites. To date, irradiation is not accepted by the organic industry. Also approvals for irradiation of some selected food products (e.g., almond) have not been accepted by many countries, such as EU, Japan, and Taiwan [31].

### 2.3. Controlled Atmosphere Storage

Controlled atmosphere, a disinfestation technology wherein the normal composition of atmospheric air, that is, 21% O_2_, 0.03% CO_2_, and 78% nitrogen, is altered appropriately for disinfestation process [26]. An atmosphere containing more than 35% CO_2_ known as carbon dioxide atmosphere and the atmosphere containing less than 1% oxygen, that is, low-oxygen atmosphere are lethal to insects. Controlled atmospheres are mainly based on the establishment of a low-oxygen environment which kills pests. The oxygen levels vary between 0% and 2%. It can be applied in airtight environments ranging from 1 M^3^ to 1000 M^3^ depending upon food commodity. Insects in all stages are eliminated because of the lack of oxygen which causes the insect to dry out and suffocate. This controlled atmosphere (CA) storage has been shown to be promising in creating lethal conditions for insects and fungi in stored food commodities. Annis and Morton [36] studied the effect of 15% to 100% CO_2_ on developmental stages of insects in wheat at 25°C and 60% RH. They found that pupae were the most tolerant stages for all CO_2_ concentrations and eggs were the only stages with 100% mortality at 20%  CO_2_ for less than 30 days. Gunasekaran and Rajendran [37] also found that the pupae stages were the most tolerant stages when exposed to different concentrations of CO_2_. Controlled atmospheres are always being seen as average alternative such as longer treatment time, usability and availability, and being not suitable for dealing with high level of infestation.

### 2.4. Conventional Heat Treatment

Compared to the use of chemical methods, heat disinfestation has the potential for high market acceptance because of being residue-free. Most research focused on using hot air (80°C to 100°C) for disinfestation of food grains and showed satisfactory results. Thermal treatment methods using hot air/hot water alone or in combination with cold or controlled-atmosphere (CA) storage conditions have been investigated extensively for disinfestation of number of stored commodities [28, 38–55]. The hot air used to increase the temperature of the food product lies above the thermal limits of survival of the pest/insect. Heat disinfestation treatments are relatively easy to apply, leave no chemical residues, and may offer some fungicidal activity. There are a number of factors that affect the mortality of insects when exposed to hot air such as duration of exposure, temperature, species, and stage. Ideal temperatures for growth, reproduction, and movement for most stored product insects are between 25°C and 35°C [56]. Many insect larvae can bore into the center of fruits, nuts, seeds, or kernels, so the center of the commodity must be heated to lethal temperatures. Reported heating times for fruit center to reach the desired maximum temperatures range from 23 min for cherries to 6 h for apples [48, 49]. Conventional heating consists of heat transfer from the heating medium to the fruit surface and then conductive heat transfer from the surface to the center. A common difficulty with hot air or water heating methods is the slow rate of heat transfer resulting in long treatment time [57]. Prolonged heating is proved to be detrimental to the quality of final products such as peel browning, pitting, poor color development, and abnormal softening and may not be practical in industrial applications ([58], cited by [59]). There were reports of damage to grapefruits and mangos when exposed to forced air at 46°C for 3.75 h and 45°C for 1.8 h, respectively ([60, 61] cited by [48]). Flavor and appearance of air-heated grapefruits at 46°C for 3 h were inferior to those of unexposed fruits [62]. Surface browning in avocados was observed when heated with hot air at 43°C for 3.5 h [63] and in apples when exposed to hot water treatment at 46°C for 45 min [64]. A long exposure time requirement also causes alterations to flavor compounds [65]. The low heating rates also may increase the thermotolerance of the few insects [66–68] which was caused by the induction of heat shock proteins in insects during sublethal thermal conditions [69]. It is also important to determine the time-temperature combinations that result in 100% mortality for each insect over relatively large range of temperature. Sometimes temperature and time combinations required to kill the target insects may exceed those that reduce the crop nutrients, germination, or shelf life [70]. The disadvantages associated with conventional heat treatment method stimulated further studies on the possible use of dielectric heating for controlling stored-grain insects.

### 2.5. Dielectric Heating

Dielectric heating which covers both radio frequency (RF) and microwave (MW) has been investigated for insect control in foods [71]. Radio frequency (RF) heating is akin to microwave heating but utilizes another part of the electromagnetic spectrum. The frequency used for MW is 2450 MHz or 915 MHz while for RF the frequency is of 13, 27, or 40 MHz [48]. The effects of RF and MW energy are generally believed to be mainly thermal in nature [72]. Most agricultural products that considered dielectric material can store electric energy and convert electric energy into heat [49]. As the wavelength of RF heating frequencies is 22 to 360 times as more than that of the microwave frequencies, this allows RF energy to penetrate dielectric materials more deeply than microwaves. Many studies have explored the use of RF heating for control of insects in agricultural commodities [31, 47, 49, 73–82]. Researchers have reported the acceptable product quality after treating nuts and rice with RF energy to control insect infestation [48, 76–78, 83–85]. Recently, Wang et al. [81] studied postharvest disinfestation treatments for chickpeas and lentils using RF energy and also reported acceptable product quality. But the radio frequency heating is particularly useful when applied to institutional-sized packaged food products because of its deep penetration [86, 87].

Thermal treatment methods involving microwave radiation have extensively been investigated by several researchers as an alternative method of killing insects. Microwave quarantine method seems to have a great potential as an alternative method of killing insects in stored grain because of several advantages such as the control of all developmental stages of storage pests, having no chemical residues on the food product, having minimal impact on the environment, and providing rapid heating [28, 88–90]. Insects are unlikely to develop resistance to this treatment [91]. This electromagnetic energy (MW and RF) interacts directly with the product's interior to quickly raise the center temperature [47–49]. Microwave radiation not only kills insects by the dielectric heat induced within them but also affects the reproduction of survivors [92]. Microwave radiation, with good penetrability, can kill pests existing inside or outside grain kernels [93]. This paper brings the research initiatives especially on microwave (MW) heating for their potential use for disinfestation of stored food grains.

#### 2.5.1. Microwave Heating

Microwaves are electromagnetic waves with frequencies ranging from about 300 MHz to 300 GHz and corresponding wavelengths from 1 to 0.001 m [94]. In electromagnetic spectrum, microwaves lie between radio frequencies and infrared radiation. Microwave heating is based on the transformation of alternating electromagnetic field energy into thermal energy by affecting polar molecules of a material. Many molecules in food (such as water and fat) are electric dipoles, meaning that they have a positive charge at one end and a negative charge at the other, and therefore they rotate as they try to align themselves with the alternating electric field induced by the microwave beam. The rapid movement of the bipolar molecules creates friction and results in heat dissipation in the material exposed to the microwave radiation. Microwave heating is most efficient on liquid water and much less on fats and sugars (which have less molecular dipole moment) [95]. The most important characteristic of microwave heating is volumetric heating which is different from conventional heating. The electromagnetic energy directly interacts with commodities to raise the interior temperature and significantly reduce treatment times as compared to conventional hot-water immersion and heated air methods. Conventional heating occurs by convection or conduction where heat must diffuse from the surface of the material. Volumetric heating means that materials can absorb microwave energy directly and internally and convert it into heat. The power generated in a material is proportional to the frequency of the source, the dielectric loss of the material, and the square of the field strength within it. The conversion of microwave energy to heat is expressed by the following equation [96]:

(1) $$\begin{array}{c} p=2\pi E^{2}f\varepsilon^{\prime \prime }\varepsilon_{0}V, \end{array}$$

where *p*: power, W, *E*: the electric field strength, V/m, *f*: the frequency, Hz, *ε*_0_: the permittivity of free space, F/m, *ε*′′: the dielectric loss factor, and *V*: volume of the material, m^3^.

Dielectric properties of food depend on composition, temperature, bulk density, and microwave frequency. Since the influence of a dielectric depends on the amount of mass interacting with the electromagnetic fields, the mass per unit volume, or density, will also have an effect on the dielectric properties. This is especially notable with particulate dielectrics such as food grains. For granular and particulate materials, both the dielectric constant and loss factor tend to increase linearly with increasing bulk density of the materials especially in lower moisture materials such as cereal grains [97].

#### 2.5.2. Microwave Disinfestation

The use of microwaves for disinfestation is based on the dielectric heating effect produced in grain, which is a relatively poor conductor of electricity. An attractive feature of the insect control using the microwave energy is that the insects are heated at a faster rate than the product they infest because of high moisture content of insects. So, it is possible to heat the insects to a lethal temperature because of their high moisture content while leaving the drier foodstuff unaffected or slightly warm [12]. Raising the temperature of infested materials by any means can be used to control insects if the infested product can tolerate the temperature levels that are necessary to kill the insects. There has been a lot of research on microwave disinfestation of cereals especially wheat [33, 98–104] and of some other food materials such as nuts [28], corn [105], pulses [106, 107], and cherries [89]. Exposure to microwave energy could cause physical injuries and reduced reproduction rates in surviving insects [75]. 

Feasibility of microwave disinfestation of insect pests has been explored by Andreuccetti et al. [74] for woodworms and by Halverson et al. [108] for wheat, maize, and flour weevils (cited by [89]). Nelson [75] reviewed the susceptibility of various stored grain insect species to radio frequency and microwave treatments. The use of microwave energy to control insects was initiated by Webber et al. [109] and the literature prior to 1980 has been reviewed by Tilton and Vardell [98]. Researchers have reported that microwave treatment is an attractive quarantine treatment. The major advantage of microwave heating is that it interacts directly with food grains and significantly reduce the amount of time required for food grain to reach the lethal temperature for insects as compared with conventional heating methods. Based on microwave heating theory and dissipated power calculations in (1), the absorption of microwave energy is proportional to the dielectric constant and dielectric loss factor of the materials. The dielectric properties of the materials depend on the frequency of the applied electric field and the temperature of the material [110]. If the material is hygroscopic, dielectric properties also depend on the amount of water in the material [111].

The first reported measurements of insect dielectric properties were for rice weevil and flour beetle at 40 MHz frequency. The dielectric constants were 6.6 and 7.8 for rice weevil and flour beetle, respectively, and loss factor was 2.2 for both species [112–114]. Nelson [110] presented data that confirms the dielectric loss factor of the insects was found to be much higher than that of stored-grain insects. The insect permittivity data at 25°C for 2.47 GHz frequency reported by Nelson et al. [32] are shown in Table 1. Alfaifi et al. [115] have reported that the loss factor of insect pests, such as Indian meal moth (*Plodia interpunctella*) and navel orangeworm (*Amyelois transitella*), is 26 to 36 times greater than that of dried fruits. Wang et al. [28, 48, 49] have indicated preferential heating of insects when walnut kernels and codling moth were subjected to radio frequency (27 MHz) and microwave frequency (2450 MHz) heating. Similar finding was observed by Ikediala et al. [116] and Raskovan et al. [117] for insect infested grain when subjected to radio frequency heating. They have reported that the dielectric constant and dielectric loss factor for granary weevil were greater than those of host grain in frequency band from 2 to 150 MHz. The reason attributed is that the higher the water content, the higher the values of dielectric properties of a material [118] which consist of dielectric constant (*ε*′) and dielectric loss factor (*ε*′′) described by the complex relative permittivity *ε* ^*^ [115, 119]:

(2) $$\begin{array}{c} \varepsilon^{*}=\varepsilon^{\prime }-j\varepsilon^{\prime \prime }, \end{array}$$

where *j* = (−1)^0.5^, *ε*′ = real component, a measure of the ability of the material to store electromagnetic energy, and *ε*′′ = Imaginary component, a measure of the ability to dissipate electrical energy into heat.

Table 2 shows the dielectric permittivity of food when subjected to microwave heating. Hallman and Sharp [120] also summarized research on the application of radio frequency and microwave heat (electromagnetic energy) treatments to kill different pests on many postharvest food crops. Power level and exposure time of microwave have been identified as two important parameters to provide 100% insect mortality [101]. Microwave energies have been investigated for a number of food products other than cereal grains such as pulses, nuts, cherries, and dates. Recently, the effect of the microwave-heating method has been studied on disinfestations of the stored green gram seed [107]. The power level and exposure time were optimized based on insect mortality, color, and antinutrient factor with the value of power and exposure time of 800 W and 80 s, respectively. Singh et al. [106] have also studied the disinfestation of pulse beetle (adult stage) in chickpea, pigeon pea, and green gram as a function of exposure time and power level by exposing it continuously to microwave radiation (2450 MHz). The mortality of insect was found to increase with increase in microwave exposure time and power level or both. Though seed viability and germination of all these pulses were found to be affected, the cooking and milling characteristics were not affected by microwave exposure time and power level. Campana et al. [121] and Bhaskara Reddy et al. [122] that though eradication of the insects increased with the increase in microwave energy, but the seed viability, germination capacity, and seedling vigour decreased by exposure to microwave energy. Vadivambal et al. [104] and Vadivambal [33] had studied the mortality of different life stages of three common stored-grain insects, namely, *Tribolium castaneum* (Herbst), *Cryptolestes ferrugineus* (Stephens) and *Sitophilus granarius* (L.) in wheat, barley, and rye, respectively, using industrial microwave dryer operating at 2.45 GHz. Grain samples of 50 g at 14%, 16%, and 18% moisture content (wet basis) were exposed to four different power levels of 200, 300, 400, and 500 W for two exposure times of 28 and 56 s. Complete (100%) mortality was achieved for adults of all three insect species at 500 W for an exposure time of 28 s. The average temperature of wheat, barley, and rye at 500 W and 28 s was around 80°C, 71°C and 82°C, respectively. In all the cases, eggs were found to be most susceptible followed by larvae, and the least susceptible were the pupae and adults. Halverson et al. [103] had also reported that egg and young larva of all the three species were more susceptible than the pupa when microwave energy at 28 GHz frequency. The species tested were *S. granarius, T. castaneum and R. dominica*. Germination of seeds was lowered with an increase in power level or exposure time or both but there was no significant difference found in the quality characteristics of microwave-treated and control wheat, barley and rye except for a decrease in the flour yield. There were also reports by several researchers that no significant difference in the quality of grain protein, flour protein, flour yield, and loaf volume of sample was found when treated with microwave energy at which 100% mortality was obtained.

Kaasova et al. [123] studied the chemical and biochemical changes during microwave treatment of wheat and reported that an improvement in the baking quality was found at higher energy doses and higher product temperatures. The decrease in germination capacity/seedling viability was related to the final temperature and the initial moisture content of the grains. Hamid and Boulanger [124] had found that the bread making quality of wheat was affected with increase in treatment temperature when exposed to power of 1.2 kW. They reported that 70% and 100% mortality were obtained when the wheat temperature was 55°C and 65°C, respectively, while, Locatelli and Traversa [125] have reported that temperature of grain has to reach 80°C for achieving complete mortality of insects infected using microwave. In fact, most infesting biological agents do not survive over a certain temperature called lethal temperature, generally between 55°C and 60°C, which can be rapidly reached through microwave irradiation [40]. There are three temperature zones for all insects: optimum, the zone at which highest rate of development can be achieved; suboptimum, a zone below or above optimum during which insects can complete their life cycle; and thirdly lethal zone, above or below suboptimum zones when insects get killed over period of time (Table 3). But the lethal temperatures to kill insects were found to vary considerably not only with species but also with the stage of development, temperature, and relative humidity. When developing effective treatment protocols, it is essential to determine which of the targeted insects is the most heat resistant, as well as the most heat resistant life stage for each species. To accomplish this, information is required on the minimum time-temperature combinations that result in 100% mortality for each insect over a relatively large range of temperature. The thermal death kinetics for fifth-instars of Indian meal moth, codling moth, and navel orange worm have been separately reported [76, 77, 126–129]. The authors of [130] recommended 15 min exposures to microwaves, which increased temperature to 60°C to kill all insects in confectionery walnuts without adversely affecting flavor.

Halverson et al. [103] conducted experiments to determine life stages of the insect that is most susceptible to microwave energy at 28 GHz frequency. The life stages tested were egg, young larva, and pupa. Their results suggested that egg and young larva of all were always more susceptible than the pupa. Zouba et al. [131] had employed a research-scale microwave unit to investigate the mortality of insect in date and the thermal impact on the date quality. The heating characteristics of dates are highly influenced by initial moisture content. Date having various moisture contents gets heated up in a very heterogeneous manner and the energy of the microwaves seems to be preferentially absorbed by soft dates. Similar results were reported during insect control of walnuts using radio frequency treatments. The insect mortality is achieved in less than 90 s during microwave treatment without altering date quality.

#### 2.5.3. Challenges in Microwave Disinfestation

Although microwaves have potential for disinfestation applications in the grain industry, they have not been used widely due to their adverse effects on various quality parameters. The major problems associated with microwave heating are the nonuniform temperature distribution and thereby incomplete kill of microbes [132–137]. Hot spots, produced on products due to the nonuniform heating pattern of microwaves, are one of the important factors for the quality degradation of products during microwave treatment. A hot spot can be defined as a local area of very high temperature that results from the temperature dependence of material properties. Although hot spots are desirable to increase insect mortality, detrimental effects on the medium are not. Manickavasagan et al. [138] had determined the germination percentage of microwave-treated wheat samples collected from hot-spot and normal heating zones. The wheat samples having four different moisture levels (12%, 15%, 18%, and 21% wb) were subjected to different microwave treatments (100, 200, 300, 400, and 500 watt with exposure times of 28 and 56 s) in continuous industrial microwave dryer (2450 MHz). The germination percentage of the sample in the hot-spot region was significantly lower than that of the normal heating region for all moisture and power levels. Apart from germination, the other quality parameters were also found to get affected more in the hot-spot zone than the remaining bulk grain. Moreover, the generation of “hot spots” during industrial microwave heating has recently become of great concern to scientists involved in microwave research [139, 140]. The other major drawback in microwave disinfestation is the poor penetrating power of microwaves. The microwave's intensity diminishes with increased penetration. It has been reported that microwave treatment of bulk grain is not feasible when the depth is greater than 4 inch ([92] cited by [33]). Due to the limited penetration of microwave energy into foodstuff mass, it seems likely that employment of microwave radiation alone could not be considered as a promising insect control measure under field condition. Disinfestations of stored products using microwaves energy coupled with other modes of treatment can be an alternative measure in killing insects effectively, but little work has been reported on combined application. The mechanisms involved in the lethal action of microwave radiation are already understood. Microwave radiation has effects on insects such as reduction of reproductive rate, losing body weight, and malformation [75]. However, application of microwaves radiation could be limited due to insufficient penetration depth. Songping et al., [140] reported that microwaves attenuate exponentially in penetration to foodstuffs.

Combined application of microwaves with hot-air treatment/cold storage/gamma radiation could be considered as a potential measure which can help reduce stored-product insects population. The combined impact of microwave radiation and cold storage on adults was evaluated by several researchers. Ayvaz and Karabörklü [141] had reported that there is a decrease in reproductive ability and number of living adults depending on the length of the cold storage period. In general, the reduction of temperature in the environment stresses the insect, thereby, making it more susceptible to other control measures [48, 89]. The major advantage is that the cold storage can easily be coupled with microwave radiation for pest control measure. There was sufficient indication that longer microwaves energy exposure and cold storage duration could achieve better kill than shorter ones of similar power level. When the insects such as *Tribolium castaneum *H. and *Sitophilus oryzae *L. were exposed to 2450 MHz at power level of 100 W for exposure time 10 min, continuously and intermittently, the highest rate of mortality was achieved for intermittent exposure time of 10 min and 72 h of cold storage duration [142]. Valizadegan et al. [143] have also evaluated the impact of microwave radiation in conjunction with cold storage on adult insects (saw-toothed grain beetle and cigarette beetle) under laboratory conditions. The insects were exposed to 2450 MHZ at five different power levels of 0, 100, 200, 300, and 400 W for five exposure times of 0, 3, 6, 9, and 12 min. The complete control was achieved at 400 W power levels for exposure time of 12 min and 72 h cold storage period. Similar results, that is, high mortality rate, were also reported by Nasab et al. [5] when Indian meal moth eggs were exposed to power levels of 100, 300, and 500 W with 2, 4, 6, 8, and 10 min exposure duration along and then kept in cold storage conditions (4 ± 1°C) for 24 and 48 h. Combinations of microwave radiation and cold storage were found to be compatible and synergistic which provide an effective and environmentally friendly disinfestation treatment technique.

Researchers have also tried the microwave heating along with gamma irradiation to control insects in stored cereals and cereals products. El-Naggar and Mikhaiel [144] evaluated the biochemical analyses on the samples of wheat grain and flour subjected to combined microwave and gamma irradiation treatment where high mortality was obtained. There were no detectable changes in the quality of protein, fat, fiber, carbohydrates, or ash. Amjad and Akbar Anjum [145] stated that when onion seeds (*Allium cepa* L.) were exposed to various doses of gamma radiation, that is, 0, 20, 40, 80, and 100 krad, there was no significant effect on seed viability except at the highest dose (100 krad) which resulted in reduced viability. But several other researchers reported that microwaves are not suitable for the drying of wheat which is to be used as seeds, even using low power levels, unless some provisions are made to ensure uniform heating [124, 146–148]. They have concluded that bread making quality of wheat and maize get adversely affected by exposure to microwave radiation. Though microwave heating helped in reducing the power consumption in wheat milling process, the textural characteristics of the final products are made from the microwave-treated flour were found not to be acceptable. The viscosity of the flour decreased with increased exposure time because of the alteration in structure of starch and protein when exposed to microwave energy. Campana et al. [121] had also stated that the protein content was not affected but the functionality of gluten altered gradually with increasing time of exposure. The change in the functionality of gluten is because of the absence of elasticity and stretchability of the dough [121, 147].

Several researchers concluded that germination capacity was affected by exposure to microwave energy. The seed viability and germination of chickpea, pigeon pea, and green gram were also reported to be affected by microwave exposure time and power level [106]. Vadivambal et al. [149] found that microwave energy at 2450 MHz with similar heating mechanism to RF treatments might control storage insects in barley and rye but resulted in poor germination due to high sample temperatures. The decrease in germination capacity was related to the final temperature and the initial moisture content of the grains. Nelson and Stetson [150] studied the dielectric heating treatments of rice weevils in wheat at 39 and 2450 MHz and showed that the lower frequency was much more effective in killing the insects. Wang et al. [86] had concluded that differential heating of insects in walnuts does occur at 27 MHz but not at 915 MHz based on the experiments carried out on dried nuts and fruits by exposure to microwave and radio frequencies range.

Bhaskara Reddy et al. [122] studied the effect of microwave treatment on quality of wheat seeds infected with *Fusarium graminearum* (Schwabe). Their results showed that though mortality of insect increased with the microwave power level, but the seed viability and seedling vigor decreased accordingly. It has been concluded that microwave drying of wheat would not be suitable where the final products made out of flour required soft textural characteristics. Another issue with the microwave heating is the large number of factors that affect the microwave heat transfer behavior such as the thickness, the geometry, and the dielectric properties of the food. The heat capacity and the dielectric properties (dielectric constant, loss factor) change with the moisture content and temperature which complicates the microwave heating process. Cost estimates for microwave and radio frequency insect control are estimated to be around three to five times more expensive than chemical control.

## 3. Conclusion

Insects cause considerable damage to food grains with weight and nutritional losses reducing yields and market values. Postharvest phytosanitary treatments are often required to completely control insect pests before the products are moved through marketing channels to areas where the pests do not occur. Several methods have been suggested to control insect pests in agricultural commodities, including chemical fumigation, thermal treatment, ionizing radiation, cold storage, controlled atmospheres, dielectric heating, and combination treatments. Current technologies involve the use of toxic chemicals which is neither consumer friendly nor environmentally friendly and conventional thermal methods are either undesirable or cause loss of volatile components, browning, and texture change. To date, irradiation is not accepted by the organic industry. Based on the results of the microwave disinfestation studies already conducted, it is considered as safe and competitive alternative method to other quarantine methods and can avoid problems of food safety and environmental pollution. The study conducted on different food products infested with major insects says that complete mortality, that is, 100%, could be achieved using microwave energy. Although microwaves have potential for disinfesting the food products, they have not been used widely due to their adverse effects on various quality parameters. Nonuniformity of heating is one of the important factors that cause quality deterioration of food product. Though several practical means can be used to minimize non-uniform heating such as adding forced hot air to the product surface or sample movement/rotation or mixing during treatment, and so forth. Also if the microwave disinfestation is made economical, it may serve as a safe and effective alternate method of insect control. The most important factor in the development of an acceptable insect control method using microwave energy is to identify a balance between minimized thermal impact on the product quality and complete killing of the insect population. To achieve a balance between complete eradication of the insects and to maintain the product quality, further research needs to be done on large scale tests, with infested product to confirm the treatment efficacy and product quality after extended storage before this technology would provide an acceptable process for disinfestation.

## Figures and Tables

**Table 1:** Dielectric properties of insects at 20–25°C ([32] cited by [33]).

	Frequency (GHz)							
Adult insect species	0.2		2.4		9.4		20	
	*ε*′	*ε*′′	*ε*′	*ε*′′	*ε*′	*ε*′′	*ε*′	*ε*′′
*S. oryzae *	28	12	17	3	17	3	—	—
*L. decemlineata *	53	81	38	12	30	16	19	17
*S. oryzae *	42	28	32	9	25	12	18	13
*S. oryzae *	55	48	42	13	31	16	23	16
*T. castaneum *	61	56	47	15	34	19	25	19
*O. surinamensis *	70	68	53	17	40	21	28	22
*R. dominica *	63	55	43	15	34	19	25	18

*ε*′: dielectric constant, *ε*′′: dielectric loss.

**Table 2:** Dielectric properties of selected food products at 20°C.

Food product	Dielectric constant		Dielectric loss	
	915 MHz	2450 MHz	915 MHz	2450 MHz
Apple	57	54	8	10
Almond	2.1	—	2.6	—
Avocado	47	45	16	12
Banana	64	60	19	18
Carrot	59	56	18	15
Cucumber	71	69	11	12
Dates	12	—	5.7	—
Grape	69	65	15	17
Grapefruit	75	73	14	15
Lemon	73	71	15	14
Lime	72	70	18	15
Mango	64	61	12	14
Onion	61	64	12	14
Orange	73	69	14	16
Papaya	69	67	10	14
Peach	70	67	12	14
Pear	67	64	11	13
Potato	62	57	22	17
Radish	68	67	20	15
Strawberry	73	71	14	14
Walnut	3.2	—	6.4	—

Source: see [34].

**Table 3:** Response of stored-product insects to temperature [35].

Temperature (°C)	Zone	Effect
50–60	Lethal	Death in minutes
45		Death in hours

35	Suboptimum	Development stops
33–35		Development slows

25–33	Optimum	Maximum rate of development

13–25	Suboptimum	Development slows
13–20		Development stops

5	Lethal	Death in days
−10 to −5		Death in weeks to month
−25 to −15		Death in minutes, insects freeze
